# Research on the mechanism of drug–drug interaction between salvianolate injection and aspirin based on the metabolic enzyme and PK-PD model: study protocol for a PK-PD trial

**DOI:** 10.1186/s13063-018-2861-7

**Published:** 2018-09-14

**Authors:** Wantong Zhang, Baochen Zhu, Weiyi Cao, Rui Li, Shuge Wang, Rui Gao

**Affiliations:** 1 China Academy of Chinese Medicine Science, Xiyuan Hospital, Beijing, 100091 China; 2 Beijing University of Traditional Chinese Medicine, Beijing, 100029 China

**Keywords:** CHD, Salvianolate, Medicine combination, Metabolic enzymes, Drug–drug interactions, PK-PD

## Abstract

**Background:**

Coronary heart disease (CHD) is a common cardiovascular disease accounting for 10–20% mortality by heart disease worldwide. The gold standard treatment to manage CHD is aspirin, which may prevent myocardial infarction and sudden death; however, long-term use of aspirin may increase its side effects. Currently, more and more clinicians are exploring different approaches to use the right combination of medicine to enhance the efficacy and reduce side effects. Salvianolate can significantly inhibit the aggregation and activation of platelets in patients with CHD; however, its optimum combination with western medicine is not established or supported by clinical trial results.

**Methods/design:**

This trial is a prospectively planned, open-labeled, parallel-grouped, single-centered clinical trial with aggregated pharmacodynamics-pharmacokinetics (PK-PD) data. All treatment courses will last for 10 days and blood sample will be acquired before administration on days 8, 9, and 10, and after administration at 5 min, 15 min, 30 min, 45 min, 1 h, 2 h, 4 h, 8 h, 12 h, and 24 h on day 10. This trial uses PK-PD modeling to provide a description of the concentration–effect relationship and an estimate of pharmacological potency of the medicine. The primary outcome will be changes in aspirin esterase and catechol-o-methyltransferase (COMT) activity at different blood concentrations to determine the PK-PD characteristics of the combination of salvianolate and aspirin, followed by analysis of the correlation between exposure level and pharmacodynamic index of the medicines.

**Discussion:**

This trial will aim to evaluate the relationship between changes in the pharmacokinetics and therapeutic effect index in the combined use of salvianolate and aspirin. It also discusses the possible mechanism of medicine combination in the treatment for CHD and provides an experimental basis for a clinically rational medicine combination.

**Trial registration:**

ClinicalTrials.gov, NCT03306550. Registered on 9 October 2017. ClinicalTrials.gov https://register.clinicaltrials.gov/prs/app/action/SelectProtocol?sid=S0007D8H&selectaction=Edit&uid=U0003QY8&ts=2&cx=oiuc9g

**Electronic supplementary material:**

The online version of this article (10.1186/s13063-018-2861-7) contains supplementary material, which is available to authorized users.

## Background

Nowadays, cardiovascular disease has become the most important disease affecting human health [1] ; the mortality of coronary heart disease (CHD) accounts for 10–20% of total mortality by heart disease worldwide. Guidelines for the diagnosis and treatment of CHD indicate that the main objective of drug therapy for CHD is to prevent myocardial infarction and sudden death and the first choice of treatment is aspirin. Aspirin can effectively inhibit platelet aggregation and prevent blood coagulation and thrombosis [2]. However, its long-term use can increase the risk of gastric mucosa injury, bleeding, hemolysis, and hematopoietic dysfunction [3]. Therefore, more and more clinicians are exploring different approaches to use the right combination of medicine to enhance efficacy and reduce side effects. However, the optimum combination of Chinese and western medicine is not established or supported by clinical trial results; it remains unclear whether the interaction between Chinese and western medicine leads to interactions in one or more stages of pharmacodynamics and whether the interactions are synergistic or antagonistic in nature. Moreover, it is challenging to determine the appropriate dosage and route of administration to achieve the maximum clinical efficacy of the combined application of Chinese and western medicine. These are all scientific questions that need to be studied.

### Aspirin

Aspirin is among the most commonly used drugs worldwide. Its widespread use is attributed to the high prevalence of ischemic heart disease and cerebrovascular accidents, along with its proven efficacy in preventing them. Clinical trials show that antiplatelet therapy with aspirin reduces the risk of myocardial infarction, stroke, and vascular death. Aspirin can prevent platelet aggregation because it leads to decreased thromboxane A2 (TXA2) through the inhibition of cyclooxygenase (COX) combined with removed acetyl [4, 5]. The whole process deacetylates by several kinds of esterase in digestive juice and blood after oral administration. Therefore, the curative effect of aspirin may rely on its deacetylation rate in the human body. Aspirin’s curative effect may decline if it hydrolyzes too rapidly in the digestive juice and blood [6].

### Salvianolate injection

Salvianolate injection is a listed injection composed mainly of magnesium lithospermate, which constitutes > 80%, and magnesium acetate homologs. Salvianolate regulates blood circulation, blood stasis, and pulse [7]. It is used for stable angina, a class I and II CHD with mild and moderate symptoms. Recently, a favorable clinical effect has been observed in the treatment of stable angina by injection of salvianolate combined with western medicine. It is widely used in combination with aspirin, isosorbide dinitrate, calcium antagonists, ACEI, and ARB. Many clinical findings show that the combination of salvianolate injection and routine western medicine for treatment of CHD has better clinical efficacy than only medication [8].

Salvianolate can significantly inhibit the aggregation and activation of platelets in patients with unstable angina pectoris, improve microcirculation, and prevent microthrombus formation [9, 10]. It prevents platelet aggregation by reducing the platelet aggregation rate, inhibiting p-selectin expression, and reducing matrix metalloproteinase-9 activity. In CHD patients treated with clopidogrel and aspirin, it can further inhibit the expression of platelet activation complexes membrane glycoprotein GP IIb/IIIa and CD62p [11]. In vitro experiments confirmed that salvianolate inhibits platelet activation and aggregation by inhibiting platelet PDE activity and directly antagonizing P2Y12 receptor [12].

### PK-PD

The PK-PD model comprises pharmacokinetics (PK) and pharmacodynamics (PD). This model has become increasingly important, especially in preclinical trials, to support drug discovery and determine optimal dosing strategies. The PK-PD model can provide a description of the concentration–effect relationship and an estimate of pharmacological potency of the drug. This methodology allows us to describe time dependence, discriminate inter-subject variability, and perform risk assessments at an individual level [13–15].

### Why is this study needed now?

Traditional Chinese medicine (TCM) has a long history of usage in China and is also widely used as alternative medicine worldwide [16, 17]. A survey shows that approximately 1.5 million adults in the United States use one or more kinds of herbal medicine while regularly taking prescription medications [18]. Meanwhile, several clinics in the UK use TCM. However, it is unclear whether drug–drug interaction (DDI) mechanisms between TCM and western medicine limit the application of TCM [19, 20]. Therefore, it is necessary to determine the DDI between TCM and western medicine. DDI can be divided into three groups: pharmacokinetics DDI; pharmacodynamics DDI; and in vitro DDI. Pharmacokinetics DDI generally involves the interaction of drugs in the process of absorption, distribution, metabolism, and excretion. TCM, which contains many bioactive ingredients, may engage in a synergistic or antagonistic interaction with other medicines. In this study, we focus on pharmacokinetics DDI between salvianolate and aspirin. Through the establishment of the PK-PD model, we aim to evaluate the relationship between the change of pharmacokinetics and therapeutic effect index in the combined use of salvianolate and aspirin. Finally, we discuss the possible mechanism of medicine combination in the treatment for CHD and provide an experimental basis for a clinically rational medicine combination.

## Objectives

The primary objective of this study is to determine the influence of salvianolate on the activity of aspirin esterase and that of aspirin on the activity of salvianolate metabolic enzyme.

The secondary objective is to determine the PK-PD characteristics of the salvianolate and aspirin combination and analyze the correlation between the exposure level and pharmacodynamic index of the medicines.

## Methods/design

The trial is conducted in accordance with the ethical principles laid down in the Declaration of Helsinki (as revised in 1983), the Guideline for Good Clinical Practice (China, 2003 and Exposure draft in 2017), and local laws. Written informed consent was obtained from all patients enrolled after a complete and extensive description of the study and before any trial-related procedures were conducted. The trial was approved by the ethics committee of Xiyuan Hospital (Additional file 1).

### Study design

**Fig. 1:**
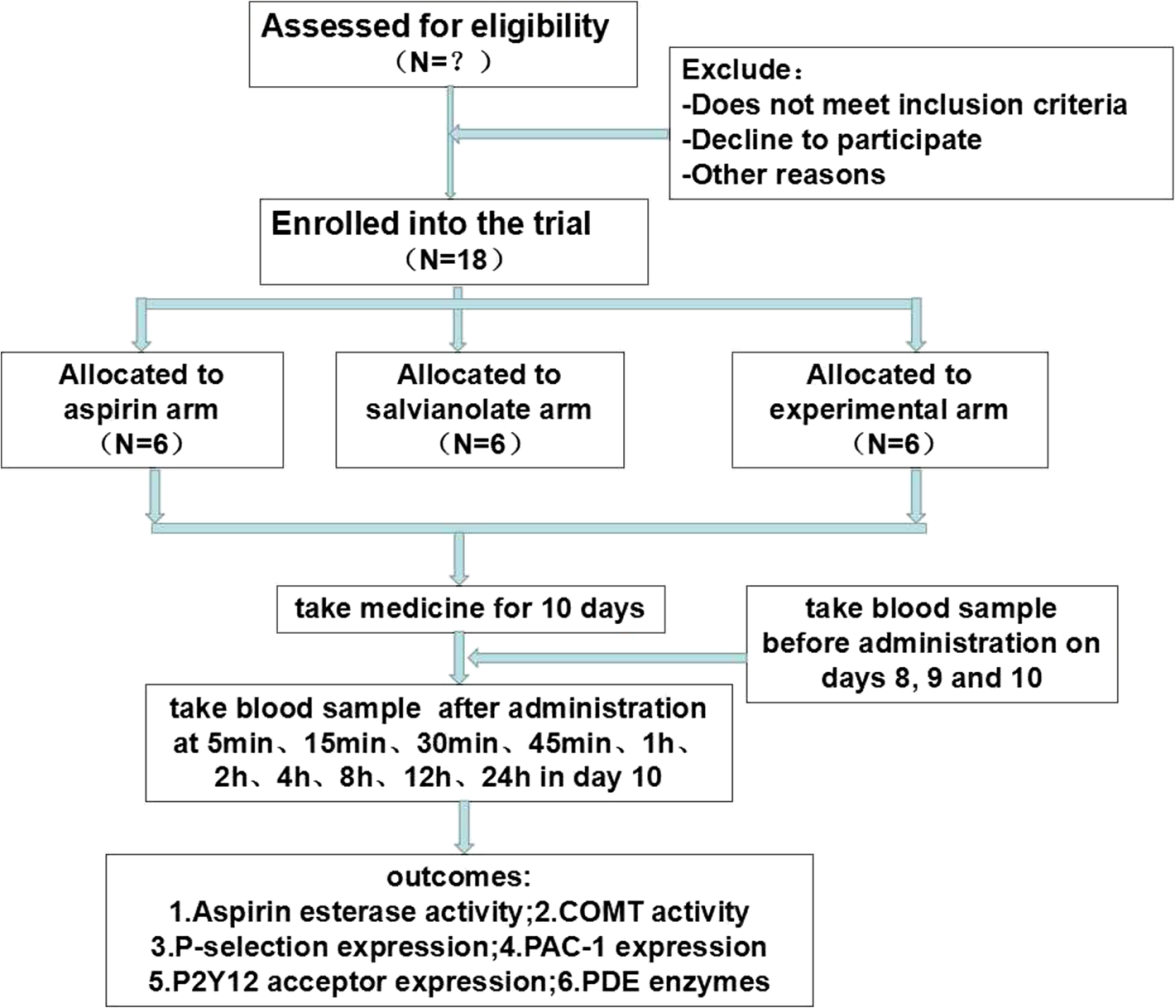
ᅟ

**Fig. 2:**
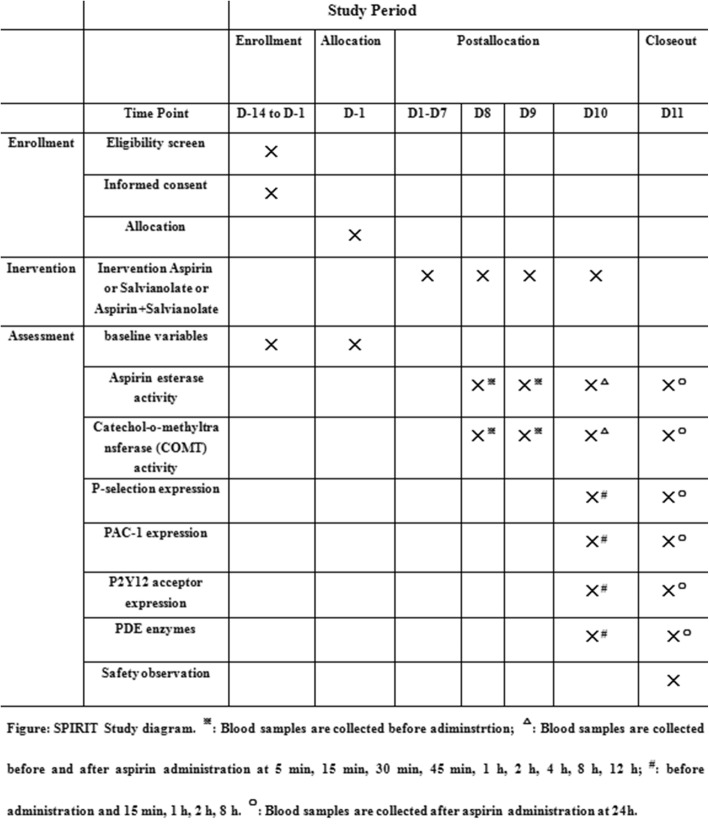
SPIRIT figure

We will conduct a prospectively planned, open-labeled, parallel-grouped, single-centered clinical trial with aggregated PK-PD data (Fig. 1). Eighteen participants will be randomly allocated into three arms: participants in the aspirin arm will be administered with aspirin (100 mg QD) orally; participants in the salvianolate injection arm will be injected with salvianolate (200 mg + 5% glucose injection 250 mL, i.v.); and participants in the experimental arm will be administered with aspirin (100 mg QD) orally and injected with salvianolate (200 mg + 5% glucose injection 250 mL, i.v.). All treatment courses will last for 10 days. Blood samples will be acquired before administration on days 8, 9, and 10, and after administration at 5 min, 15 min, 30 min, 45 min, 1 h, 2 h, 4 h, 8 h, 12 h, and 24 h on day 10 (Fig. 2).

### Study setting

The trial site will be outpatient care of the Cardiology Department at Xiyuan Hospital in China. Staff at all relevant clinics will be informed about the availability of the trial and they will refer participants to the study investigators. Intravenous therapy will be conducted in the emergency department at Xiyuan Hospital. Participants will be admitted to the hospital on day 9 and will complete the sampling period on days 10 and 11. Clinical biological samples will be sent to a pharmacokinetics laboratory for detection and pharmacokinetic data analysis.

### Study participants

To be included in the trial, participants must: (1) be aged 35–65 years; (2) meet the diagnostic criteria of CHD; (3) meet the diagnostic criteria of stable angina pectoris; (4) meet the diagnostic criteria of blood stasis; (5) meet the diagnostic criteria of grade I–II angina according to the Canadian Cardiovascular Society (CCS); (6) take regular aspirin or salvianolate injection within one month prior; and (7) be informed of the study and voluntarily sign an informed consent.

Exclusion criteria include: (1) severe heart disease (acute myocardial infarction or acute myocardial infarction in six months), severe cardiopulmonary dysfunction (e.g. cardiac function II); (2) poorly controlled hypertension (systolic pressure > 160 mmHg; or diastolic pressure > 100 mmHg); (3) diabetes; (4) severe primary diseases, such as liver and renal hematopoietic system damage, liver function (ALT ≥ 2 × ULN, AST ≥ 2 × ULN), kidney function (Cr > 1.0 × ULN), or nervous and mental disorder; (5) pregnant, breast-feeding, and menstruating women, as well as women planning pregnancy within three months; (6) having participated in clinical trials in the last three months; (7) underwent surgery or had hemorrhagic tendency in the last four weeks; (8) drug allergy history or with allergic constitution; (9) mental or physical disorders; (10) poor compliance or unsuitability for this clinical trial by investigator’s judgment.

Termination criteria include:

Participant termination

1. Serious adverse events which affect the normal life of participants or obvious abnormal value of the laboratory tests appear in the process of trial, in which case the researchers should decide to terminate the trial and immediately notify the sponsor.
2. The researchers believe that it is necessary to stop the individuals from continuing to participate in the trial because of medical, safety, or regulatory and GCP considerations.

Trial termination

1. Half of the participants have mild adverse events.
2. The researchers believe that it is necessary to abort the test for medical, safety, or regulatory purposes and for GCP.

### Interventions

1. Aspirin enteric-coated tablets (100 mg, QD) (Bayer Healthcare Co. Ltd.): an anti-platelet medicine widely used in the treatment for cardiovascular disease.
2. Salvianolate injection (200 mg + 5% glucose injection 250 mL, i.v.) (Shanghai Green Valley Pharmaceutical Co. Ltd.): the main ingredient of the salvianolate injection is salvianolic acid B (SAB), which is extracted from the root of red salvia. Red salvia is used in TCM for promoting blood circulation to counter blood stasis, which is clinically effective in the treatment for cardiovascular disease.

The detail of dose and duration of the medication is illustrated in Table 1.

**Table 1:** Dose and duration of medication

Group	Medication	Dose and method	Duration (days)
Aspirin group	Aspirin	100 mg QD, oral	10
Salvianolate group	Salvianolate	200 mg + 5% glucose 250 mL, i.v. injection	10
Experimental group	Salvianolate + aspirin	100 mg QD oral aspirin and 200 mg + 5% glucose 250 mL i.v. salvianolate injection	10

### Combination

1. Participants are not allowed to take any kind of TCM or western medicine with pharmacological effects of activating blood circulation, platelet aggregation, and anti-coagulation or belonging to the non-steroidal anti-inflammatory class.
2. In case of angina pectoris, participants can take isosorbide mononitrate after approval by researchers. It is permitted to take other medicines that are not metabolized through aspirin esterase and catechins-O-methyl transferase (COMT) and the record the combination.

### Randomization

This is a single-center clinical trial of three groups. The sample size is 18 and all participants will be distributed 1:1:1. The randomization code will be developed by the study statistician. The trial cases were divided into three processing groups. The random numbers of “trial cases” was produced by SAS random procedure: they were arranged vertically by block and randomly coded. The randomized coding of test cases was the same as that of the experimental medicine. When a participant has been enrolled, the research pharmacy will randomize the individual based on the master randomization list.

### Sample collection

Blood sampling will be conducted in three groups:

1. First group (aspirin group) sampling process
  The participants will take aspirin before breakfast for 10 consecutive days. Blood samples will be collected before aspirin administration on days 8 and 9. After the participants take aspirin on an empty stomach on the morning of day 10, blood samples will be collected from the elbow vein before administration and at 5 min, 15 min, 30 min, 45 min, 1 h, 2 h, 4 h, 8 h, 12 h, and 24 h after administration. The participants should maintain a half-berth for 2 h during non-blood sampling time, during which they will be monitored for blood pressure and heart rate.
2. Second group (salvianolate group) sampling process
  The participants will receive salvianolate injection in the emergency room of Xiyuan Hospital after a meal for 10 consecutive days. Blood samples will be collected before administration on days 8 and 9. The participants will be injected with salvianolate on an empty stomach on the morning of day 10. The injections will be dropped by 1.5 h ± 15 min. Blood samples will be collected from the elbow vein before injection and at 5 min, 15 min, 30 min, 45 min, 1 h, 2 h, 4 h, 8 h, 12 h, and 24 h after administration. The participants should maintain a half-berth for 2 h during non-blood sampling time, during which they will be monitored for blood pressure and heart rate.
3. Third group (experimental group) sampling process
  The participants take aspirin before breakfast followed by salvianolate injection in the emergency room of Xiyuan Hospital after a meal for 10 consecutive days. Blood samples will be collected before administration on days 8 and 9. Salvianolate will be injected on an empty stomach on the morning of day 10, 2 min after which the participants will take aspirin with 200 mL warm water. The injections will be dropped by 1.5 h ± 15 min. The blood samples will be collected from the elbow vein before administration and at 5 min, 15 min, 30 min, 45 min, 1 h, 2 h, 4 h, 8 h, 12 h, and 24 h after administration. The participants will be able to maintain a half-berth for 2 h during non-blood sampling time, during which they will be monitored for blood pressure and heart rate.

## Proposed trial outcome measures

### Primary outcome

1. Change in aspirin esterase activity at different blood concentrations: biological analysis will be conducted by liquid chromatography tandem mass spectrometry (LC-MS/MS) to detect aspirin and salicylic aspirin and salicylic acid: CSA/C(ASA + SA). (Time frame: blood samples are acquired before aspirin administration on days 8 and 9. On day 10, blood samples are acquired before and after aspirin administration at 5 min, 15 min, 30 min, 45 min, 1 h, 2 h, 4 h, 8 h, 12 h, and 24 h.)
2. Change in COMT activity at different blood concentrations: COMT activity is the main metabolic enzymes of SAB. A kit was used to detect COMT activity. (Time frame: blood samples are collected before injection on days 8 and 9. On day 10, blood samples are collected before and after injection at 5 min, 15 min, 30 min, 45 min, 1 h, 2 h, 4 h, 8 h, 12 h, and 24 h.)

### Secondary outcome

1. Change in P-selection expression (platelet activation-dependent granule membrane) at different time points: flow cytometry is used to detect the P-selection expression on platelets at different time points. The influence of aspirin/salvianolate on platelet interaction adhesion during drug concentration-time curves is analyzed. (Time frame: blood samples are acquired on day 10 before administration and 15 min, 1 h, 2 h, 8 h, and 24 h after administration.)
2. Change in PAC-1 expression (platelet-associated complement) on activated platelets at different time points: flow cytometry is used to detect the PAC-1 expression on platelets at different time points. The influence of aspirin/salvianolate on platelet aggregation during drug concentration-time curves is analyzed. (Time frame: blood sample is collected on day 10 before administration and 15 min, 1 h, 2 h, 8 h, and 24 h after administration.)
3. Change in P2Y12 acceptor expression on platelets at different time points (P2Y12 acceptor is a receptor for ADP and ATP coupled to G-proteins that inhibit the adenylyl cyclase second messenger system): flow cytometry is used to detect the P2Y12 acceptor expression on platelets at different time points. The influence of aspirin/salvianolate on ADP- or ATP-activated platelets aggregation during drug concentration-time curves is analyzed. (Time frame: blood samples are acquired on day 10 before administration and 15 min, 1 h, 2 h, 8 h, and 24 h after administration.)
4. Change in phosphodiesterase (PDE) enzyme expression on platelets at different time points: ELISA is used to detect the PDE enzyme expression at different time points. The influence of aspirin/salvianolate on platelets activated by PDE enzymes during drug concentration-time curves is analyzed. (Time frame: blood samples are collected on day 10 before administration and 15 min, 1 h, 2 h, 8 h, and 24 h after administration.)

### Safety observation

1. Blood routine;
2. Serum biochemistry: AST, ALT, ALP, GGT, TBIL, DBIL, IBIL, GLU, BUN, K+, Na+, Cl-, Ca2+;
3. Urine routine;
4. ECG.

### Adverse events

Each participant will receive safety monitoring throughout the trial. All adverse events will be forwarded to the Ethics Committee and Office of clinical trials institution. They will review all documented harms during the study and adjudicate them with regards to causality.

## Withdrawal

Patients who are lost to follow-up, have bad compliance, or use other medicine that will influence the result will be withdrawn. If a participant chooses to withdraw, a withdrawal report form will be filled out to record assessments and reason(s) for withdrawal.

## Statistical analysis plan

### Method validation

1. Selectivity

The chromatogram of standard sample, blank sample, additional sample, and post-administration sample should be established.

(2) The matrix effect, lowest limit of quantification, and stability should be tested.
(3) Standard curve and its correlated coefficients should be tested.
(4) Intra- and inter-day precision and accuracy of the method defined as the relative standard deviation (RSD) were evaluated by analyzing QC samples at three different concentrations in six replicates on the same day and three consecutive validation days [21].

### PK index

#### Aspirin esterase activity

Using data from the blood sample analysis (CSA/C(ASA + SA)), we will compare the results of the three arms by ANOVA analysis.

#### Catechol-o-methyltransferase (COMT) activity

We will use the column graph method. The results of the experimental, salvianolate, and aspirin arms will be compared by ANOVA analysis.

#### PD index

The results of the experimental, salvianolate, and aspirin arm will be compared by ANOVA analysis.

## Interim safety analysis

The DSMB comprises clinical physicians, clinical trial methodology experts, statistical experts, members of the ethics committee, and clinical pharmacists. They carry out risk assessment and safety analysis according to program termination criteria.

## Discussion

A combination of salvianolate and aspirin may provide an effective and safe therapeutic option for patients with CHD; however, its DDI mechanism remains unclear. We investigate this inquiry by applying a PK-PD model. Research and clinical practice become intimately intertwined on the ground of PK-PD trials. We apply typical research instruments (e.g. randomization, control) to achieve the ultimate clinical objective, to reliably and empirically determine the best therapeutic choice for patients, and also meet the aims of innovative and timely research. This study establishes a PK-PD model to determine the PK-PD characteristics of the salvianolate and aspirin combination and analyze the correlation between exposure level and pharmacodynamic index of the medicines.

Metabolic enzymes play an important role in pharmacokinetics DDI. The main metabolic enzyme is CYP450, which is essential for the metabolism of many drugs. According to statistics, CYP450 isoforms are responsible for the oxidative metabolism of approximately 85% of the marketed drugs [22]. However, neither aspirin nor salvianolate injection has any relation with CYP450. Aspirin is hydrolyzed by several kinds of esterases [23–25] and the main active component of salvianolate, SAB, is metabolized by COMT. These enzymes might be the key point of pharmacokinetics DDI between aspirin and salvianolate injection. In this case, we plan to use ELISA to measure the activity of COMT and a formula containing aspirin and its metabolite, salicylic acid, to evaluate the activity of esterase. In summary, we analyze the pharmacokinetics DDI of salvianolate and aspirin through COMT and esterase, respectively, to determine the influence between the metabolic processes of each medicine. This research also explores the functional mechanism of the two medicines on the treatment of CHD. It is the trend to establish PK-PD based on functional mechanism. The function of salvianolate and aspirin is related to the inhibition of platelet aggregation and blood coagulation. Thus, we choose P-selection, PAC-1, and P2Y12 acceptor and PDE enzymes as efficacy indexes to explore their correlation with drug concentration.

Ultimately, this research, in its entirety, will provide an approach of filling the gap between science and practice, supplying evidence of DDI, and facilitating the use of medicine combination in the treatment of CHD.

## Trial status

Recruitment began in April 2018.

## Additional file

Additional file 1: SPIRIT 2013 checklist. (DOC 108 kb)

## Abbreviations

CHD: Coronary heart disease
COMT: Catechol-O-methyltransferase
COX: Cyclooxygenase
DDI: Drug–drug interaction
PAC-1: Platelet-associated complement
PDE: Phosphodiesterase
PK-PD: Pharmacodynamics-pharmacokinetics
P-selection expression: Platelet activation-dependent granule membrane
RSD: Relative standard deviation
TCM: Traditional Chinese medicine
TXA2: Thromboxane A2
